# The ventral habenulae of zebrafish develop in prosomere 2 dependent on Tcf7l2 function

**DOI:** 10.1186/1749-8104-8-19

**Published:** 2013-09-25

**Authors:** Carlo A Beretta, Nicolas Dross, Peter Bankhead, Matthias Carl

**Affiliations:** 1 Department of Cell and Molecular Biology, Medical Faculty Mannheim, Heidelberg University, Ludolf-Krehl-Strasse 13-17, Mannheim 68167, Germany; 2 Heidelberg University, COS and Nikon Imaging Center at the University of Heidelberg, Bioquant, Heidelberg 69120, Germany

**Keywords:** Habenula, Tcf, Zebrafish, Time-lapse imaging, Photoconversion, PSmOrange, Neural network, Asymmetry

## Abstract

**Background:**

The conserved habenular neural circuit relays cognitive information from the forebrain into the ventral mid- and hindbrain. In zebrafish, the bilaterally formed habenulae in the dorsal diencephalon are made up of the asymmetric dorsal and symmetric ventral habenular nuclei, which are homologous to the medial and lateral nuclei respectively, in mammals. These structures have been implicated in various behaviors related to the serotonergic/dopaminergic neurotransmitter system. The dorsal habenulae develop adjacent to the medially positioned pineal complex. Their precursors differentiate into two main neuronal subpopulations which differ in size across brain hemispheres as signals from left-sided parapineal cells influence their differentiation program. Unlike the dorsal habenulae and despite their importance, the ventral habenulae have been poorly studied. It is not known which genetic programs underlie their development and why they are formed symmetrically, unlike the dorsal habenulae. A main reason for this lack of knowledge is that the vHb origin has remained elusive to date.

**Results:**

To address these questions, we applied long-term 2-photon microscopy time-lapse analysis of habenular neural circuit development combined with depth color coding in a transgenic line, labeling all main components of the network. Additional laser ablations and cell tracking experiments using the photoconvertible PSmOrange system in GFP transgenic fish show that the ventral habenulae develop in prosomere 2, posterior and lateral to the dorsal habenulae in the dorsal thalamus. Mutant analysis demonstrates that the ventral habenular nuclei only develop in the presence of functional Tcf7l2, a downstream modulator of the Wnt signaling cascade. Consistently, photoconverted thalamic *tcf7l2*^*exl/exl*^ mutant cells do not contribute to habenula formation.

**Conclusions:**

We show *in vivo* that dorsal and ventral habenulae develop in different regions of prosomere 2. In the process of ventral habenula formation, functional *tcf7l2* gene activity is required and in its absence, ventral habenular neurons do not develop. Influenced by signals from parapineal cells, dorsal habenular neurons differentiate at a time at which ventral habenular cells are still on their way towards their final destination. Thus, our finding may provide a simple explanation as to why only neuronal populations of the dorsal habenulae differ in size across brain hemispheres.

## Background

The habenulae are part of the conserved dorsal diencephalic conduction system and connect the forebrain with the ventral mid- and hindbrain via axon bundles named fasciculi retroflexi [1]. In teleosts, these efferent axons derive from the bilateral dorsal (dHb) and ventral habenular (vHb) nuclei, which innervate the interpeduncular nucleus (IPN) and the median raphe (MR), respectively [2-5]. Consistent with the function of network components in the regulation of a number of neurotransmitters, the habenulae have been implicated in a range of behaviors and cognitive functions, and aberrant habenular network function has been correlated with pathophysiological syndromes such as depression and schizophrenia in mammalian model systems and humans [6-10]. An additional intriguing hallmark of the system is the distinct asymmetric character of the dHb across the left-right axis in many vertebrates with respect to neuroanatomy, expression of various molecules and connectivity patterns [3,4,11-14]. Also in mammals, subtle size differences between the left and right medial habenulae, the structure homologous to the dHb of teleosts [2], have been described [15]. In particular these asymmetric features have led to researchers investigating this part of the habenulae as a model for functional lateralization of the brain, common to all vertebrates [16,17]. To assess this fundamental aspect in neuroscience, a considerable amount of work has been dedicated to elucidating the genetic cascades underlying asymmetric dHb development, to be able to manipulate the network and study the consequences [11,18,19]. In contrast, the analysis of vHb development has been largely neglected. For instance, it is known that dHb neurons differentiate from pools of precursor cells adjacent to the pineal complex on the left and right [20], but nothing is known about the origin of the vHb. This, however, comes as a surprise as the lateral habenulae, the mammalian part of the habenulae homologous to the vHb in teleosts [2], is a prominent component of the serotonergic system modulating for instance aggressive behavior [21-23]. Moreover, this part of the habenulae was found to be a key-regulator of the reward system [24-27]. Interestingly, the lateral habenulae in albino mice exhibit a right-sided lateralization [28], but the functional importance has not been reported.

To find out about vHb origin and development, we used 2-photon microscopy time-lapse imaging and followed habenular neural circuit development for four consecutive days in transgenic zebrafish embryos. We found evidence that vHb neurons originate in the thalamic-epithalamic part of prosomere 2, and during development come to lie adjacent to the forming dorsal habenulae. We validated our observation by laser cell ablations and by tracking cells using the photoconvertible protein PSmOrange [29] in the GFP transgenic background. This system also allowed us to demonstrate that vHb neuron development crucially relies on the Wnt pathway downstream modulator Tcf7l2.

## Results and discussion

### Long-term imaging suggests the intermingling of habenular neurons derived from different clusters in the dorsal diencephalon

**Figure 1:**
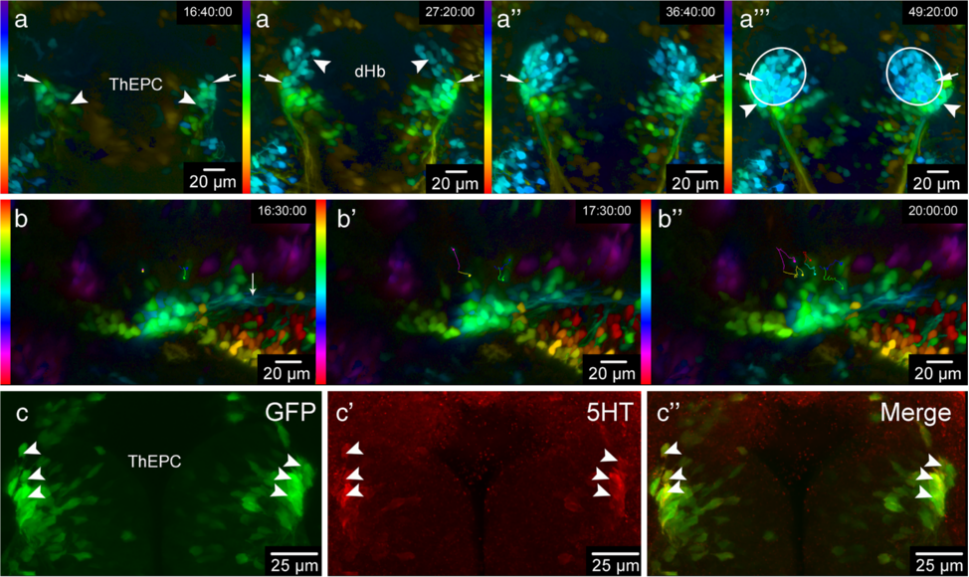
**A subpopulation of thalamic-epithalamic early projecting cluster (ThEPC) cells may contribute to the habenulae. (a-a”’)** Color code MIP, dorsal views with anterior to the top of the habenula area in living *Et*(−*1*.*0otpa*:*mmGFP*)*hd1* embryos at 46 hpf, 57 hpf, 68 hpf and 79 hpf (left to right; see Additional file 1: Movie S1). LUT (look up table) shows the z color code with a z-depth value of 300 μm. Arrows indicate the most anterior ThEPC cells, which appear to end up in the lateral part of the habenulae. **(a)** Arrowheads indicate the left and right ThEPCs. **(a’)** Arrowheads indicate the left and the right dHb domain. **(a”’)** Arrowheads highlight ThEPC cells, which remain outside the habenulae (circles). **(b-b”)** Spectrum MIP, dorsal views with anterior to the left of left-sided ThEPC cells at 48 hpf, 49 hpf and 52 hpf (see Additional file 2: Movie S2). LUT (look up table) shows the z spectrum with a z-depth value of 200 μm. Dots and lines indicate examples of manually tracked dividing cells. **(b)** Arrow indicates ThEPC axons. **(c-c”)** Dorsal view of the thalamic area, MIP, anterior to the top, of a 48 hpf *Et*(−*1*.*0otpa*:*mmGFP*)*hd1* embryo stained for 5HT. From left to right: GFP, red and merged channels. Arrowheads highlight some co-labeled GFP/5HT positive neurons. The gamma was adjusted to a value of 0.80 **(b,c)**. dHb, dorsal habenula; ThEPC, thalamic-epithalamic early projection cluster; MIP, maximum intensity projection.

**Figure 2:**
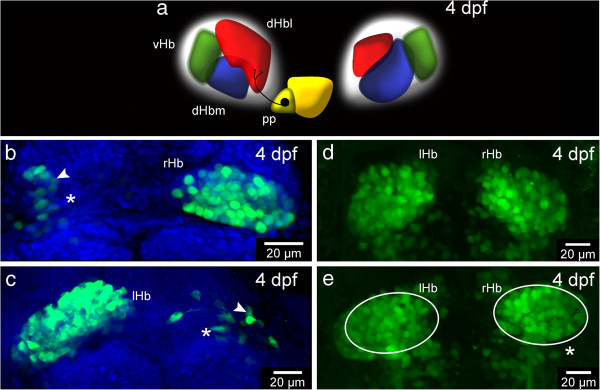
**Ablation experiments suggest a contribution of thalamic-epithalamic early projecting cluster (ThEPC) cells to the habenulae. (a)** Habenular nuclei at 4 dpf. dHbl, lateral dorsal habenula (red); dHbm, medial dorsal habenula (blue); vHb, ventral habenula (green); P, pineal (yellow); pp, parapineal (dark yellow). **(b-e)** MIP, anterior to the top, dorsal views of the habenular nuclei of 4 dpf *Et*(−*1*.*0otpa*:*mmGFP*)*hd1* embryos, after (b) left and **(c)** right dHb ablation at 2 dpf. Asterisks mark the ablated side and white arrowheads highlight neurons located in the lateral habenular domain. The nuclei (in blue) are labeled with Sytox Orange. **(d,e)** Habenula formation at 4 dpf in **(d)** not ablated and in **(e)** ThEPC ablated *Et*(−*1*.*0otpa*:*mmGFP*)*hd1* embryos. Asterisk marks the ablated side. Same sized ellipses highlight the habenula size differences. The gamma was adjusted to a value of 0.80. lHb, left habenula; rHb, right habenula; ThEPC, thalamic-epithalamic early projecting cluster.

All habenular nuclei and their efferent projections targeting the interpeduncular nucleus (IPN) and median raphe (MR) are labeled in *Et(−1.0otpa:mmGFP)hd1* transgenic embryos (unpublished data). In addition to GFP expression starting at 43 hpf in developing dorsal habenular (dHb) neurons in close proximity to *cxcr4b* expressing habenular precursor cells ([20] and unpublished data), a bilateral cell cluster in prosomere 2 (hereafter named thalamic-epithalamic early projecting cluster or ThEPC), posterior and lateral to them, expresses GFP (Figure 1a,a’). Measuring the distance of ThEPCs and dHb neurons at 48 hpf in 2D, we find them separated by about 40 μm. Performing long-term 2-photon microscopy (2-PM) high-resolution imaging of habenular neural circuit development combined with depth color coding, we noticed that the most anterior ThEPC cells appear to come to lie adjacent to dHb neurons over time in a lateral position (Figure 1a-a”’ and Additional file 1: Movie S1). This part of the established habenulae has previously been shown to contain ventral habenula (vHb) neurons at this developmental stage (Figure 2a) [2]. To find out more about the ThEPC cell composition, we imaged ThEPC development in *Et(−1.0otpa:mmGFP*)*hd1* transgenic embryos with high magnification, starting before the onset of GFP expression in dHb cells at 43 hours post fertilization (hpf) (Additional file 2: Movie S2). While some ThEPC neurons project axons (Figure 1b and Additional file 2: Movie S2), some other cells of this cluster still divide between 48 and 52 hpf (Figure 1b-b”). Intriguingly, we found that a number of ThEPC neurons express the serotonergic marker 5HT at 2 days post fertilization (dpf), suggesting that the clusters consist of mixed cell populations (Figure 1c-c”). vHb neurons are part of the serotonergic system, which exhibits a striking asymmetry in salmon species [2,30,31]. Although we did not observe any differences in the number of 5HT expressing neurons across the brain at this developmental stage, our findings led us speculate that a subpopulation of thalamic neurons might contribute to the vHb.

### Ventral and dorsal habenular neurons originate in different areas of prosomere 2

To further support this idea, we unilaterally ablated the entire developing dHb in *Et*(−*1*.*0otpa*:*mmGFP*)*hd1* transgenic embryos after the onset of GFP expression at 53 hpf (Additional file 3: Figure S1a-b’) and investigated GFP expression in the habenulae two days later. At this time, dHb cells and ThEPC cells can be well distinguished (Additional file 3: Figure S1a inset) and the missing IPN innervation by axons from the ablated dHb served as a control for their complete absence. If the origin of the vHb differs from the dHb, we would expect to see some GFP positive neurons in the lateral part of the habenula on the ablated side. Indeed, two days after ablation, green neurons were detected in this area independent of the side of ablation (Figure 2a-c). For further evidence, we next conducted the converse experiment and unilaterally ablated one ThEPC at the onset of GFP expression in *Et* (−*1*.*0otpa*:*mmGFP*)*hd1* transgenic embryos (Additional file 4: Figure S2). Consistent with our hypothesis that some ThEPC neurons contribute to the habenulae, this ablation resulted in the development of a smaller habenula on the ablated side compared to the habenula on the non-ablated side (Figure 2d,e).

**Figure 3:**
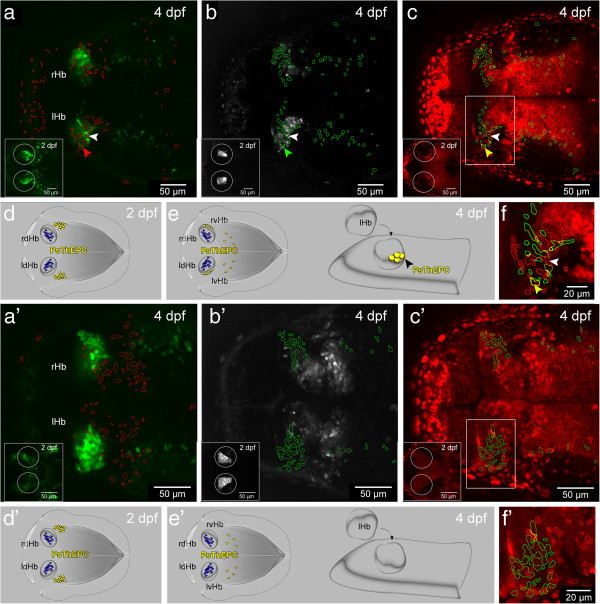
**A population of thalamic cells forms the ventral habenula (vHb) in the presence of Tcf7l2 function. (a-c’)** Dorsal views, anterior to the left, of a slice from a stack showing 4 dpf **(a-c)***Et*(−*1*.*0otpa*:*mmGFP*)*hd1* and **(a’-c’)***tcf7l2*^*exl*/*exl*^ x *Et*(−*1*.*0otpa*:*mmGFP*)*hd1* embryos after photoconversion of H2B-PSmOrange positive cells in the thalamus at 2 dpf (insets). Circles highlight the area of photoconversion. All targeted cells express the photoconverted protein and are in the centre of the circles. The gamma was adjusted to a value between 0.60 and 0.90. **(a-a’)** Red ROIs show the position of the photoconverted cells in the green channel. **(b-b’)** Green ROIs show the location of GFP positive cells in the far-red channel. **(c-c’)** Red and green ROIs were combined in the red channel to visualize the position of GFP photoconverted ThEPC neurons in the habenular nuclei in yellow. Box indicates the region shown in f and f’ with higher magnification for clarity. **(a-c,f)** Colored arrowheads mark a photoconverted ThEPC derived GFP positive cell in the habenula. White arrowheads mark a photoconverted GFP negative cell in the habenula. **(a’-c’,f’)** Photoconverted cells are not found in the habenulae of *tcf7l2*^*exl*/*exl*^ mutants. **(d-e’)** Model showing the contribution of some ThEPC cells (yellow) to the vHb in wild type embryos but not in *tcf7l2*^*exl*/*exl*^ mutants. dHb cells are marked in blue. lHb, left habenula; lvHb, left ventral habenula; Ps, photoconverted; rHb, right habenula; rvHb, right ventral habenula; ROI, region of interest; ThEPC, thalamic-epithalamic early projecting cluster.

Even though our time-lapse, marker analysis and ablation experiments strongly suggested that dHb and vHb spatial origins differ, we set out to further corroborate our finding by tracking thalamic cells over time. We used the photoconvertible fluorescent H2B-PSmOrange protein, which changes its emission from orange to far-red upon blue light treatment [29]. We find that the photoconverted protein is stable for at least three days in zebrafish and is an excellent tool for cell tracking in GFP transgenic fish (Figure 3a-c). We used the GFP positive ThEPC cells as a thalamic landmark and photoconverted the protein in, and closely around these cells at 2 dpf (Figure 3a-c insets) and analyzed their position in the brain two days later (Figure 3a-c). Thus, cells carrying the photoconverted protein at 4 dpf were derived from the thalamic area irrespectively of whether they co-expressed GFP. We developed an automated co-localization macro for ImageJ to unambiguously identify cells that express the photoconverted far-red fluorescent protein or co-express GFP and the photoconverted far-red fluorescent protein in the context of the habenular morphology highlighted by non-photoconverted orange fluorescent protein in the cell nuclei. We find on average 14 cells expressing the photoconverted protein in a vHb typical latero-ventral position in the habenulae (Figure 3a-c,f, Additional file 5: Figure S3 and Additional file 6: Movie S3). On average, eight of these photoconverted cells co-expressed GFP and between five and eight GFP negative photoconverted cells were located in the vHb in their immediate vicinity (n = 8). This indicated that unlike dHb neurons, which originate from precursor cells adjacent to the pineal complex on the left and right [20], some, if not all, vHb neurons originate from a more postero-lateral region of prosomere 2 (Figure 3d,e).

This finding may constitute a simple explanation as to why the vHb form symmetrically across the midline. The dHb consist of the lateral and the medial sub-nuclei, which are asymmetrically large in size between the left and right sides of the brain (Figure 2a). The differentiation of dHb precursor cells into neurons of these sub-nuclei is critically influenced by yet unknown signals from a group of parapineal cells, which during development migrate out from the anteriormost part of the pineal organ and are only present on the left side of the brain adjacent to developing dHb neurons [3,12,32-34]. The removal of these parapineal cells between 24 and 28 hpf results in the formation of symmetric dHb due to an increased number of precursors differentiating into medial dHb neurons at the expense of lateral dHb neurons on the left side of the brain. At this time, the future vHb cells are still on their way towards their final destination and may therefore not receive any parapineal cell signals. Alternatively, vHb precursors could receive the parapineal cell signals, which, however, do not influence their differentiation program due to different intrinsic properties between vHb and dHb precursors. This scenario is less likely as the dHb progenitors, which are influenced by parapineal cell signals, are in close vicinity of the parapineal cells [11], suggesting that the signal is acting over only a relatively short distance.

### Tcf7l2 function is essential for ventral habenula development

**Figure 4:**
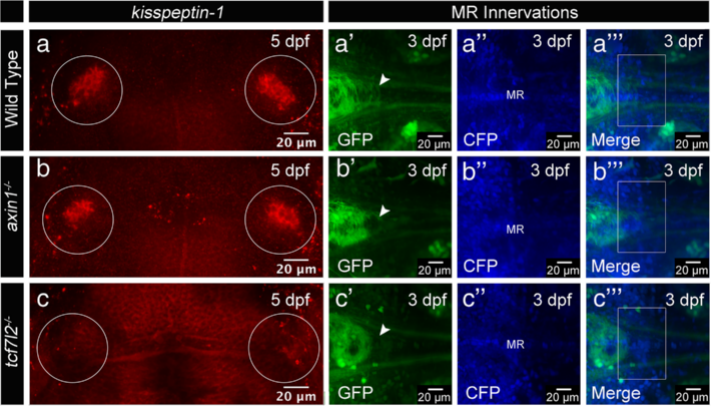
**Tcf7l2 function is required for ventral habenula (vHb) development. (a**-**c)** Dorsal views, anterior to the top. *kisspeptin*-*1* expression in the vHb (circle) is not affected by the upregulation of Wnt/beta-catenin signaling in **(b)***axin1* mutant embryos, but absent in **(c)***tcf7l2*^*exl*/*exl*^ mutants. **(a’-c”’)** MIPs, anterior to the left, dorsal views of axons innervating the MR in *Et*(*1*.*0otpa*:*mmGFP*)*hd1* transgenic embryos. H2A-CFP labels the cell nuclei. **(b’-b”’)** Upregulation of Wnt signaling in *axin1* mutants does not affect MR innervation by vHb efferent axons. **(c’-c”’)** In *tcf7l2*^*exl*/*exl*^ mutants, the MR are not innervated. White arrowheads and white boxes highlight the location of MR innervating axons, absent in **(c’-c”’)**. The gamma was adjusted to values between 0.70 and 0.80. vHb, ventral habenula; MR, median raphe.

The upregulation of Wnt signaling in *axin1* mutants [35] causes the formation of symmetric dHb with right-sided character similar to parapineal cell ablated embryos [18,36]. The parapineal cells often migrate to the left side of the mutant brains but their signaling cues do not influence dHb cell development in the presence of upregulated Wnt signaling. Thus, Wnt activity appears to influence the communication between pineal complex and dHb. The involvement of Wnt signaling in dHb development and the established Wnt function in thalamus development [37-39] prompted us to investigate whether this pathway may also be involved in vHb development. However, we found that impaired Axin1 function does not affect the formation of the vHb as judged by the expression of the vHb cell marker *kisspeptin*-*1* (*kiss*-*1*) [40] and the innervation of the vHb axon targets, the MR (Figure 4a-a”’, b-b”’).

To further elucidate the potential role of Wnt signaling in vHb development, we investigated embryos mutant for the Wnt signaling component *tcf7l2*[41], a gene widely expressed in the diencephalon [42]. *Tcf7l2*^*exl*/*exl*^ mutant embryos exhibit no morphological alterations for up to six weeks of development [41]. We find that *kiss*-*1* expression is lost in *tcf7l2*^*exl*/*exl*^ mutants and the MR are not innervated by vHb axons (Figure 4a-a”’, c-c”’). Moreover, photoconverted H2B-PSmOrange positive ThEPC cells did not localize in the vHb at 4 dpf in the mutants (Figure 3a’-c, f’ and Additional file 5: Figure S3). These data indicate that Tcf7l2 function is required for the formation of the vHb. At the same time, our mutant analysis confirms the thalamic origin of vHb cells.

Tcf7l2 is a context dependent transcriptional downstream modulator of the Wnt signaling pathway [43]. However, in the light of our finding that only defective Tcf7l2 function and not impaired Axin1 activity affects vHb development, we can only speculate that Tcf7l2 acts as an activator of Wnt signaling in the context of vHb development. In this scenario, only active Tcf7l2 mediated Wnt signaling but not the Axin1 mediated suppression of Wnt signaling is important for vHb development.

It is still an outstanding question as to which Wnt ligands are involved in habenula development. A number of ligands such as *Wnt1*, *Wnt3a*, *Wnt7* genes and *Wnt8b* are expressed in, or in close proximity to, developing dHb neurons [18,44] and may regulate the development of dHb neurons. *Wnt3a* is also expressed in the roof plate of prosomere 2 and influences the patterning and neurogenesis in the thalamus and the related thalamic mantle zone, which expresses downstream genes of Wnt signaling and harbors post-mitotic thalamic neurons [39]. It will be intriguing to determine whether indeed Wnt3a signaling influences vHb neuron development.

## Conclusions

Using long-term time-lapse techniques, computational color coding of z-positions, laser cell ablations and cell tracking methods, we show that the dHb and vHb originate in different areas of prosomere 2. Furthermore, we identify *tcf7l2* as the first gene essential for vHb development. The vHb has important roles modulating the serotonergic system in vertebrates and its impairment in humans has various consequences ranging from mood fluctuations to schizophrenia and suicidal behavior [7,21]. Intriguingly, *tcf7l2* has also been linked to schizophrenia [45,46] and in the light of our results it is tempting to speculate about a connection between Tcf7l2 function, vHb development and schizophrenia.

## Methods

### Fish maintenance

The zebrafish lines AB/TL, *Et*(−*1*.*0otpa*:*mmGFP*)*hd1*, *tcf7l2*^*exl*/*exl*^ x *Et*(−*1*.*0otpa*:*mmGFP*)*hd1* and *axin1*^*tm235*^ x *Et*(−*1*.*0otpa*:*mmGFP*)*hd1* were maintained and bred according to standard procedures [47]. All animal procedures were approved by the Regierungspräsidium Karlsruhe (permit AZ 35–9185.81/G-60/12). To inhibit pigmentation, embryos were incubated in 0.2 mM PTU.

### *tcf7l2*^*exl*/*exl*^ x *Et*(−*1*.*0otpa*:*mmGFP*)*hd1* genotyping

Genomic DNA was extracted from tails of *tcf7l2*^*exl*/*exl*^ x *Et*(−*1*.*0otpa*:*mmGFP*)*hd1* incross derived embryos. A *tcf7l2* DNA fragment was amplified using the following primers: PrFw 5′-AAAATGCCGCAGCTGAAC-3′ and PrRw 5′-CAACAACACGGTGCATCG-3′. The point mutated base pair was identified by BsajI digestion.

### Immunohistochemistry and *in situ* hybridization

*In situ* hybridizations, antibody labelings and fluorescent *in situ* labelings were carried out as described [18,48-50]. Antibodies and fluophores used: rabbit anti-GFP (1:1000, Torrey Pines, CA, USA), Rabbit anti-5HT (1:1000, Sigma, Taufkirchen, Germany), Alexa Fluor 488 (1:250, Molecular Probes, Darmstadt, Germany), Alexa Fluor 647 (1:200, Molecular Probes, Darmstadt, Germany).

Whole mount *in situ* hybridization was performed with *kiss*-*1*.

The nuclei were labeled incubating embryos with Sytox Orange (1:100.000, Invitrogen, Darmstadt, Germany) in PBS, 0.8% Triton X-100, 1.0% BSA for 30 minutes.

### 2-PM laser ablation

2-PM laser ablations of cells were performed using a Nikon 16× water dipping LWD objective (NA 0.80; Düsseldorf, Germany) on a multi photon LaVision BioTec TriM Scope (Bielefeld, Germany) mounted on an upright Nikon FN-1 microscope (Düsseldorf, Germany). Wavelength was set to 740 nm and laser power to 200 to 300 mW at the objective output.

### Subcloning of H2B-PSmOrange and injection of mRNAs

The pH2B-PSmOrange construct [29] (Addgene plasmid #31920) was subcloned into the pCS2+ vector using the blunted XbaI restriction site. 260 pg *H2B*-*PSmOrange* mRNA were injected into one cell stage embryos. 80% (n = 150) of injected zebrafish embryos showed strong nuclear H2B-PSmOrange protein expression for at least 4 days.

To label cell nuclei, 130 pg *H2A*-*CFP* mRNA was injected.

### H2B-PSmOrange photoconversion and analysis

For H2B-PSmOrange photoconversion, the photoconversion tool from the NIS-Elements AR software (Düsseldorf, Germany) was used: 17 to 20 mW of 488 nm excitation laser power; 1/2 scan speed frequency; 20 to 28 stimulation runs. 50 hpf *H2B*-*PSmOrange* mRNA injected embryos were imaged before and after photoconversion using the sequential scanning mode for the 488, 561 and 637 nm channels. To determine the position of ThEPC cells, a z-stack was acquired at 4 dpf with z-intervals of 1.0 to 2.0 μm.

The stacks were analyzed with our automatic ImageJ macro (Additional file 7). To highlight the regions of interest (ROIs), different automatic thresholds were applied in the green and far-red channel stacks after applying a 3D ‘difference of Gaussians’ filter to suppress noise and structures larger than the areas of interest. ROIs for each thresholded area were created with the Analyze Particles command, and overlapping ROIs identified and displayed in yellow.

### 2-PM setup

2-PM imaging and ablations were performed on a TriM Scope 2-photon microscope (LaVision BioTec GmbH, Bielefeld, Germany) mounted on a Nikon FN-1 upright stand (Düsseldorf, Germany) enclosed in a dark box, equipped with a femtosecond Ti:Sa laser (Chameleon Ultra II, Coherent, CA, USA), three PMTs (green channel: GaAsP, Hamamatsu; blue and red: standard, Hamamatsu, Herrsching am Ammersee, Germany) mounted on the ultrasensitive port next to the objective for increased sensitivity. Water-dipping objectives (16×, 0.8 numerical aperture (NA), long working distance (LWD), Nikon and 60×, 1.0 NA NIR Apo, Nikon, Düsseldorf, Germany) were used for image acquisition.

### Confocal laser-scan microscopy (CLSM) and image analysis

For CLSM, embryos were embedded in 1.0% low melting agarose in a glass bottom dish (MatTek, MA, USA or LabTek, CA, USA). Confocal images and stacks were acquired with a Nikon A1R (Düsseldorf, Germany) using a 20× air objective lens (NA 0.75); z-stack intervals were between 1.0 μm and 2.0 μm.

3D reconstructions, stack analysis and image adjustments were performed using the software Fiji, NIS-Element AR, Düsseldorf, Germany, Adobe Photoshop CS4, München, Germany and ImageJ (NIH).

## Abbreviations

2-PM: Two-photon microscopy; CLSM: Confocal laser-scan microscopy; dHb: Dorsal habenula; dpf: Days post fertilization; GFP: Green fluorescent protein; hpf: Hours post fertilization; IPN: Interpeduncular nucleus; LUT: Look up table; MIP: Maximum intensity projection; MR: Median raphe; PMT: Photomultiplier tube; ROI: Region of interest; Tcf: t-cell specific factor; ThEPC: Thalamic-epithalamic early projecting cluster; vHb: Ventral habenula.

## Competing interests

The authors declare that they have no competing interests.

## Authors’ contributions

CB designed and performed all experiments. CB and ND performed the time-lapse imaging and photoconversion experiments. PB developed the color code. MC designed and conceived the study and wrote the manuscript, which has been approved by all authors.

## Supplementary Material

Additional file 1 **Movie S1.** Long-term 2-PM time-lapse imaging reveals intermingling cells of different clusters, related to Figure 1. Dorsal view with anterior to the left, colour coded MIP obtained from a total z-height of 300 µm focussed on the diencephalon in a *Et(-1.0otpa:mmGFP)hd1* transgenic embryo. Time-lapse between 32 hpf and 92 dpf. The LUT shows the Z colour code table according to the depth of each stack. The stacks were acquired every 40 minutes with z-steps of 1.0 µm. Gamma was adjusted to a value of 0.70 for display purposes. White arrowheads mark the ThEPCs (arrows mark anterior ThEPC neurons), lHb, rHb, and dHb; red and green arrowheads highlight the second and the third cluster of projecting neurons, respectively. d, dorsal; Hb, habenula; IPN, interpeduncular nucleus; l, left; MIP, Maximum Intensity Projection; r, right; Tec, optic tectum; ThEPC, thalamic-epithalamic early projecting cluster. Click here for file

Additional file 2 **Movie S2.** The ThEPCs are composed of mixed populations of dividing cells and postmitotic neurons, related to Figure 1. Dorsal view with anterior to the left, Colour Code MIP obtained from a total Z-height of 200 µm. High magnification time-lapse focussed on the ThEPC located in the left brain hemisphere between 32 hpf and 52 hpf. The colour code spectrum table was used to highlight the depth of each stack. Stacks were acquired every 10 minutes with z-steps of 1.0 μm. Laser power correction was used to compensate for increasing depth (gamma = 0.60). The manual tracking identifies dividing ThEPC cells, while other GFP positive neurons send out efferent projections. MIP, Maximum Intensity Projection; ThEPC, thalamic-epithalamic early projecting cluster. Click here for file

Additional file 3: Figure S1 Ablation of dHb cells at 53 hpf, related to Figure 2. Click here for file

Additional file 4: Figure S2 Ablation of ThEPC cells at 32 hpf, related to Figure 2. Click here for file

Additional file 5: Figure S3 Photoconverted thalamic cells attach to the dHb, related to Figure 3. Click here for file

Additional file 6 **Movie S3.** A population of ThEPC cells contribute to the vHb and is absent in embryos mutant for *tcf7l2*, related to Figure 3. Dorsal view with anterior to the left, z-stacks of *Et(-1.0otpa:mmGFP)hd1 and tcf7l2-/- x Et(-1.0otpa:mmGFP)hd1* transgenic embryos at 4 dpf after photoconversion of the H2B-PSmOrange protein in the ThEPC region at 2 dpf. Each stack was acquired using the sequential scanning mode with z-steps between 1.0 μm and 2.0 μm. Gamma was adjusted for display purposes for each channel to values between 0.60 and 0.90. Colocalisation studies were performed using an automatic ImageJ macro for Fiji to identify the GFP positive, photoconverted ThEPC cells in the entire z-stack. The red channel is used to visualise the habenular morphology due to the nuclear expression of non-photoconverted H2B-PSmOrange protein at 4 dpf. Red ROIs display the location of the photoswitched positive cells, while green ROIs show the position of the GPF positive cells. Red and green ROIs were combined to display the position of ThEPC cells expressing both GFP and the photoconverted protein in yellow. Minor corrections and compression were performed using the software iMovie and Wondershare Video Converter Ultimate. Hb, habenula; ROI, region of interest; ThEPC, thalamic-epithalamic early projecting cluster; v, ventral. Click here for file

Additional file 7 Automated macro for ImageJ (see Methods). Click here for file
