# Supplementary material for: The ventral habenulae of zebrafish develop in prosomere 2 dependent on Tcf7l2 function
Source: Neural Dev. 2013 Sep 25;8:19. doi: 10.1186/1749-8104-8-19 (PMC3827927; doi:10.1186/1749-8104-8-19)
Supplement: Additional file 3: Figure S1 — Ablation of dHb cells at 53 hpf, related to Figure 2. [file 1749-8104-8-19-S3.doc]

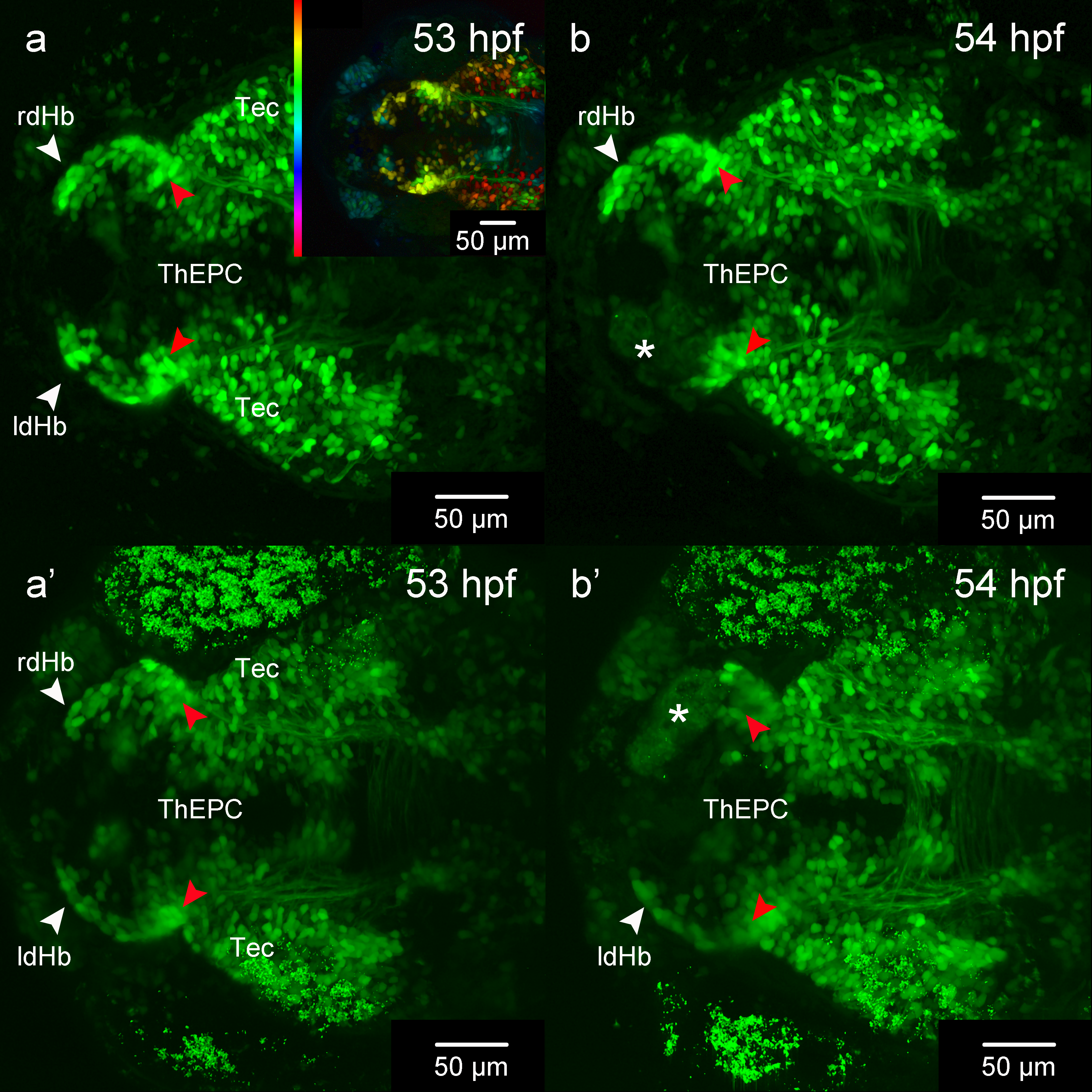


**Additional file 3: Figure S1. Ablation of dHb cells at 53 hpf, related to Figure 2.**

(a-b’) Dorsal view, anterior to the left, MIP of *Et(-1.0otpa:mmGFP)hd1* transgenic embryos before and after complete dHb cell ablation. (a-a’) White arrowheads mark the bilateral dHb nuclei before dHb ablation. Red arrowheads highlight the position of ThEPC neurons. Inset shows a spectrum LUT with a z-depth of 350 µm to highlight that dHb and ThEPC cells can be distinguished because of their different positioning along the D-V and A-P axis. Gamma was adjusted to 0.65. (b-b’) Asterisks mark the site of ablation, white arrowheads mark non-ablated dHb cells and the red arrowheads mark the ThEPC neurons. GFP expression was monitored between 2 and 4 days.

The original stacks were cropped and the gamma was corrected to 0.45 for display purposes.

d, dorsal; Hb, habenula; IPN, interpeduncular nucleus; l, left; r, right; Tec, optic tectum; ThEPC, thalamic-epithalamic early projecting cluster; v, ventral.
