# Supplementary material for: The ventral habenulae of zebrafish develop in prosomere 2 dependent on Tcf7l2 function
Source: Neural Dev. 2013 Sep 25;8:19. doi: 10.1186/1749-8104-8-19 (PMC3827927; doi:10.1186/1749-8104-8-19)
Supplement: Additional file 4: Figure S2 — Ablation of ThEPC cells at 32 hpf, related to Figure 2. [file 1749-8104-8-19-S4.doc]

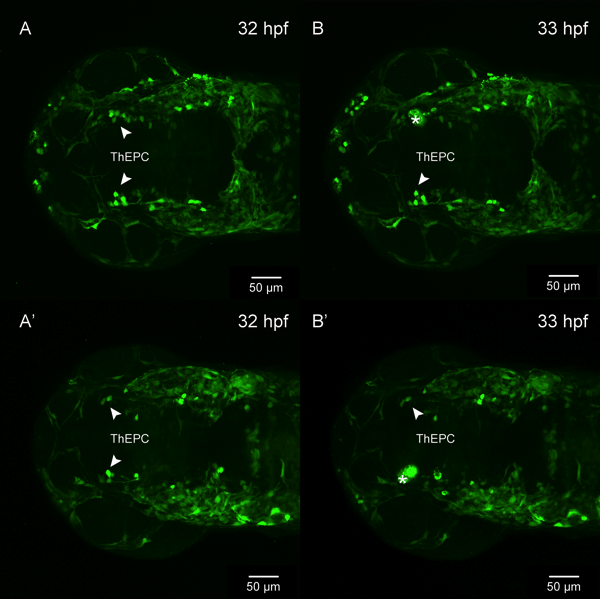


**Additional file 4: Figure S2. Ablation of ThEPC cells at 32 hpf, related to Figure 2.**

(a-b’) Dorsal view, anterior to the left, MIP of *Et(-1.0otpa:mmGFP)hd1* transgenic embryos before and after ThEPC cell ablation. (a-a’) White arrowheads mark the ThEPCs before ablation. (b-b’) Asterisks mark the site of ablation, white arrowheads mark the non-ablated ThEPC.

The original stacks were cropped and the gamma was corrected to 0.60 for display purposes.

ThEPC, thalamic-epithalamic early projecting cluster.
