# Supplementary material for: The ventral habenulae of zebrafish develop in prosomere 2 dependent on Tcf7l2 function
Source: Neural Dev. 2013 Sep 25;8:19. doi: 10.1186/1749-8104-8-19 (PMC3827927; doi:10.1186/1749-8104-8-19)
Supplement: Additional file 5: Figure S3 — Photoconverted thalamic cells attach to the dHb, related to Figure 3. [file 1749-8104-8-19-S5.doc]

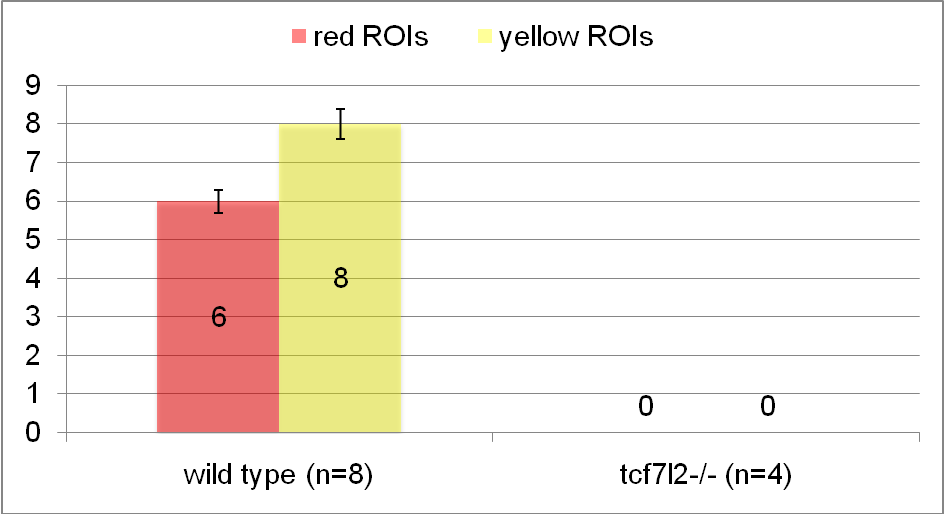


**Additional file 5: Figure S3. Photoconverted thalamic cells attach to the dHb, related to Figure 3.**

Graph shows the number of H2B-PSmOrange expressing cells, which were photoconverted at 2 dpf in the thalamus of *Et(-1.0otpa:mmGFP)hd1* transgenic wild type and genotyped *tcf7l2^exl/exl^* mutant embryos and contributed to the vHb at 4 dpf. The red column shows the number of photoconverted H2B-PSmOrange expressing cells, the yellow column represents the number of cells co-expressing also GFP. ROI, region of interest.
