# Supplementary material for: The ventral habenulae of zebrafish develop in prosomere 2 dependent on Tcf7l2 function
Source: Neural Dev. 2013 Sep 25;8:19. doi: 10.1186/1749-8104-8-19 (PMC3827927; doi:10.1186/1749-8104-8-19)
Supplement: Additional file 7 — Automated macro for ImageJ (see Methods). [file 1749-8104-8-19-S7.pdf]

```
// Colocalisation H2B-PSmOrange

setBatchMode(true);

dir_out = getDirectory("Choose the OUTPUT root directory");
if (lengthOf(dir_out) == 0) {
	exit();
}

run("Bio-Formats Importer", "color_mode=Default view=Hyperstack
stack_order=XYCZT");

idOrig = getImageID();
run("Duplicate...", "duplicate channels=1");
idCh1 = getImageID();
run("Enhance Contrast", "saturated=0.35 stack");

// Detect on Channel 1 to make ROIs
run("Duplicate...", "duplicate");
run("32-bit");
idCh1Filtered = getImageID();
run("Gaussian Blur 3D...", "x=6 y=6 z=1");
imageCalculator("Subtract stack", idCh1Filtered, idCh1);
run("Multiply...", "value=-1 stack");
run("Gaussian Blur 3D...", "x=1 y=1 z=0.5");
setAutoThreshold("Triangle dark stack");

// Analyze Particles Channel 1
// Exclude very small detections (< 5 pixels)
run("Analyze Particles...", "size=5-Infinity circularity=0.00-1.00 show=Masks add in_situ
stack");
roiManager("Show All without labels");
roiManager("Show All");
roiManager("Set Color", "green");
roiManager("Save", dir_out + "488_ROI.zip");

// Open Channel 3 and add the Channel 1 overlay
selectImage(idOrig);
run("Duplicate...", "duplicate channels=3");
idCh3 = getImageID();
roiManager("Show All without labels");
roiManager("Show All");
run("From ROI Manager");
run("Enhance Contrast", "saturated=0.35 stack");
saveAs("Tiff", dir_out + "C3_488_ROI.tif");
roiManager("Delete");

// Detect on Channel 3 to make ROIs
run("Duplicate...", "duplicate");
idCh3Filtered = getImageID();
```

```
run("32-bit");
run("Gaussian Blur 3D...", "x=16 y=16 z=1");
imageCalculator("Subtract stack", idCh3Filtered, idCh3);
run("Multiply...", "value=-1 stack");
run("Gaussian Blur 3D...", "x=1 y=1 z=0.5");
setAutoThreshold("Moments dark stack");

// Analyze Particles Channel 3
// Exclude very small detections (< 3 pixels)
run("Analyze Particles...", "size=3-Infinity circularity=0.00-1.00 show=Masks add in_situ
stack");
roiManager("Set Color", "red");
roiManager("Save", dir_out + "637_ROI.zip");

// Select Channel 1 and add the Channel 3 overlay
selectImage(idCh1);
roiManager("Show All without labels");
roiManager("Show All");
run("From ROI Manager");
saveAs("Tiff", dir_out + "C1_637_ROI.tif");
close();

// Find the detected regions overlapping in Channel 1 and Channel 3
imageCalculator("AND stack", idCh1Filtered, idCh3Filtered);
idOverlaps = idCh1Filtered;
selectImage(idOverlaps);
roiManager("Delete");
run("Analyze Particles...", "size=3-Infinity circularity=0.00-1.00 show=Nothing add
stack");
roiManager("Set Color", "yellow");
roiManager("Save", dir_out + "Overlay_ROIs.zip");
roiManager("Delete");

// Add ROI C1/C3 in C2 Channel
selectImage(idOrig);
run("Duplicate...", "duplicate channels=2");

list = getFileList(dir_out);
for (i=0; i<list.length; i++) {
	if (endsWith(list[i], ".zip"))
	roiManager("open", dir_out +list[i]);
}

roiManager("Show All without labels");
roiManager("Show All");
run("From ROI Manager");
run("Enhance Contrast", "saturated=0.35 stack");
saveAs("Tiff", dir_out + "Overlay_C2_ROIs.tif");
run("Close All");
call("java.lang.System.gc");
```

```
showStatus("Completed");
setBatchMode(false);
```
